# Antimicrobial Stewardship—a practical guide to implementation in hospitals

**DOI:** 10.1093/jacamr/dlz005

**Published:** 2019-04-08

## Abstract

**Graphical Abstract:**
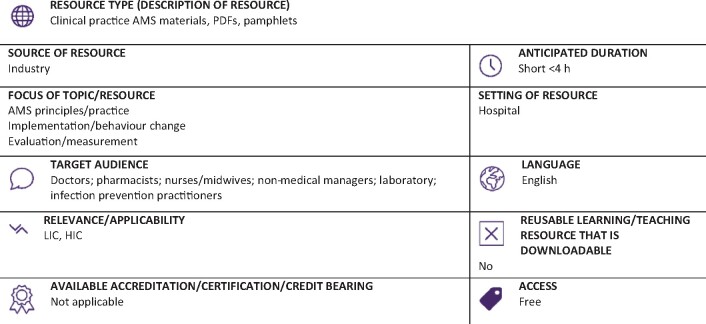

**Resource web link:** http://bsac-jac-amr.com/wp-content/uploads/2019/04/Biomer-2019-ANTIMICROBIAL-STEWARDSHIP-BOOKLET-FINAL2.pdf (Full classification scheme available at: http://bsac.org.uk/wp-content/uploads/2019/03/Educational-resource-review-classification-scheme.pdf)

**WHO region and country (World Bank):** Europe, France (LMIC, HMI, HIC)

## Peer review commentary

This is a very useful synthesis and summary of a number of key antimicrobial stewardship (AMS) resources and national recommendations from mostly developed/HIC countries, but also containing useful references and information from other parts of the world (LMICs). Recently revised and updated, it has a lot of really useful diagrams/graphs based on adaptations of data from key papers which work well to reinforce the text. The text is brief, so the entire booklet won’t take long to read through, but there are a wealth of references and pointers to additional information and resources to further support the reader. As is outlined in the title, this is directed at the hospital level, the benefits of setting up an AMS programme and how to go about implementing, monitoring and evaluating one. As would be expected from a small booklet like this, the advice is brief and at a high level, so you won’t find advice/guidance on an intravenous-to-oral switch programme or an example clinical guideline for pneumonia, but the importance of these sorts of AMS activities is explained, referenced and the user is signposted to resources to support them. If you had to implement a hospital AMS programme from scratch, with no experience of the field before this, or if you want a resource that has most of the key references for this area, this booklet would be a very good starting point.

